# Expression, Tissue Localization and Serodiagnostic Potential of *Echinococcus granulosus* Leucine Aminopeptidase

**DOI:** 10.3390/ijms19041063

**Published:** 2018-04-03

**Authors:** Maodi Wu, Min Yan, Jing Xu, Yuqing Liang, Xiaobin Gu, Yue Xie, Bo Jing, Weimin Lai, Xuerong Peng, Guangyou Yang

**Affiliations:** 1 Department of Parasitology, College of Veterinary Medicine, Sichuan Agricultural University, Chengdu 611130, China; tuzigeyao@163.com (M.W.); yanmin1201@outlook.com (M.Y.); 18502869677@163.com (J.X.); 15378204406@163.com (Y.L.); guxiaobin198225@126.com (X.G.); zhandegaokandey123@163.com (Y.X.); jingbooo@163.com (B.J.); dongxiaowei1226@outlook.com (W.L.); 2 Department of Chemistry, College of Life and Basic Science, Sichuan Agricultural University, Ya’an 625014, China; yinxiaoxiao1993@163.com

**Keywords:** leucine aminopeptidase, *Echinococcus granulosus*, indirect Enzyme-linked immunosorbent assay, immunohistochemical localization

## Abstract

*Echinococcus granulosus* is the causative agent of cystic echinococcosis (CE), a widespread parasitic zoonosis. Leucine aminopeptidases (LAPs) of the M17 peptidase family have important functions in regulating the balance of catabolism and anabolism, cell maintenance, growth and defense. In this study, we presented a bioinformatic characterization and experimentally determined the tissue distribution characteristics of *E. granulosus* LAP (Eg-LAP), and explored its potential value for diagnosis of CE in sheep based on indirect ELISA. Through fluorescence immunohistochemistry, we found that Eg-LAP was present in the tegument and hooks of PSCs, the whole germinal layer and adult worm parenchymatous tissue. Western blotting results revealed that the recombinant protein could be identified using *E. granulosus*-infected sheep serum. The diagnostic value of this recombinant protein was assessed by indirect ELISA, and compared with indirect ELISA based on hydatid fluid antigen. The sensitivity and specificity rEgLAP-ELISA were 95.8% (23/24) and 79.09% (87/110), respectively, while using hydatid fluid as antigen showed the values 41.7% (10/24) and 65.45% (72/110). This is the first report concerning leucine aminopeptidase from *E. granulosus*, and the results showed that Eg-LAP belong to M17 peptidase families, and that it is involved in important biological function of *E. granulosus*. Furthermore, rEg-LAP is appropriate for diagnosing and monitoring CE in sheep in field. Development of a rapid test using rEg-LAP to diagnose sheep CE deserves further study.

## 1. Introduction

Cystic echinococcosis (CE) is a zoonosis with worldwide distribution caused by the tapeworm *Echinococcus granulosus*; it is prevalent in northeast Africa, South America, Eastern Europe, central Asia, and western China [1,2,3,4]. World Health Organization (WHO) previously estimated that CE, results in the loss of 1–3 million disability-adjusted life years per annum [1], and almost 40 million people are at risk of infection [5]. According to incomplete statistics, over 10% of the population have hydatid cysts [6,7] or antibodies to *E. granulosus* [8] in some high-incidence areas for CE, determined by ultrasound, X-ray or ELISA, respectively. This disease also causes at least US $1.2 billion in annual losses to livestock production [9].

Traditional diagnostic methods for animal *E. granulosus* are based on necropsy in abattoirs [10,11], which commonly use judgment by macroscopic observation and have a high error rate (15.4%) [12]. There are many other ways to diagnose CE in sheep, including the flow through technique (flow anion exchange chromatography) [13], the latex agglutination test [14], and enzyme-linked immunosorbent assay (ELISA) [15,16]. These methods especially using the hydatid cyst fluid as antigens to diagnose CE of sheep are difficult to develop into commercial products. Combining effective serological methods with traditional methods (necropsy or ultrasonography issued by WHO Informal Working Group on Echinococcosis) to diagnose would make the result more credible. For human CE diagnosis, compared with native antigen B (hydatid cyst fluid antigens), recombinant antigen B had excellent specificity [17], while recombinant echinococcus protoscolex calcium binding protein using enzyme-linked immunosorbent assay (ELISA) had a sensitivity of 100% and specificity of 93.7 [18]. Indirect ELISA can be efficient and effective for early stage diagnosis of infection and the detection of infected animals [19]. For animals, a previous study of recombinant protein diagnosis for sheep CE was restricted by low sensitivity [15,20]. Therefore, to improve immunodiagnosis of CE, finding an efficient recombinant antigen is a crucial task [21].

In parasites, FgLAP can reduce the *Fasciola gigantica* burden by 60% in mice [22] and the *Fasciola hepatica* rate by 89% in sheep. Oral delivery of *Bacillus subtilis* expressing *Clonorchis sinensis* LAP2 showed that the protein could affect both systemic and local mucosal immune responses [23]. LAPs used as antigens in ELISA to diagnose several parasitic diseases have been reported [24,25,26]. However, until now, there has been no information available about the LAP of *E. granulosus*; more importantly, *E. granulosus*-specific protein antigens as potential targets for serodiagnosis of sheep CE are still scarce.

In this study, we expressed and analyzed the characteristics of a novel LAP from *E. granulosus*, and located this protein in the parasite using fluorescence immunohistochemistry. The immunogenicity of rEg-LAP was detected by Western blotting and indirect ELISA for sheep CE and was established with rEg-LAP as the coating antigen. These efforts will contribute to a better understanding of LAP, and the diagnosis of CE in sheep.

## 2. Results

### 2.1. Sequence Analysis

**Figure 1:**
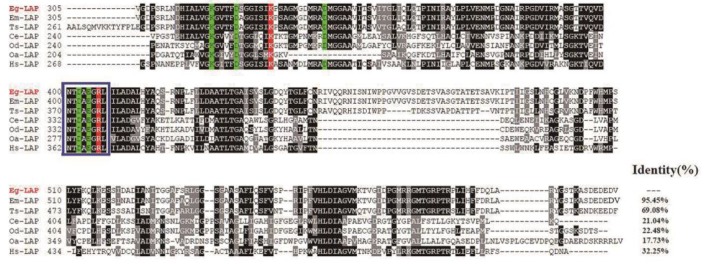
Sequence alignment analysis of Eg-LAP with homologous leucine aminopeptidases. Coherence of the deduced amino acid sequence of *Echinococcus granulosus* leucine aminopeptidase (GenBank: KX159768) with protein sequences from different species. The following sequences were retrieved from the GenBank, GeneDB and UniProt protein sequence databases: *Echinococcus multilocularis* leucine aminopeptidase (E.m-LAP) (GenBank: CDS43570.1), *Taenia solium* leucine aminopeptidase (T.s-LAP) (GeneDB: TsM_001140000), *Caenorhabditis elegans* (Ce-LAP) (GenBank: CCD73205.1), *Oesophagostomum dentatum* (Od-LAP) (UniProt: A0A0B1TS56), *Ovis aries* leucine aminopeptidase (Oa-LAP) (GenBank: XP_004014812.1) and *Homo sapiens* leucine aminopeptidase (Hs-LAP) (GenBank: AAD17527.1). Regions of high identity and similarity between leucine aminopeptidase sequences according to the Clustal X algorithm are shown as black and gray columns, respectively. The M17 family signature octapeptide NTDAEGRL sequence is marked with a blue box. The actin-binding motifs are in white letters with red backgrounds; the metal binding sites are in black letters with green backgrounds. The percentage homology of Eg-LAP with other species is shown at the end of the alignments.

**Figure 2:**
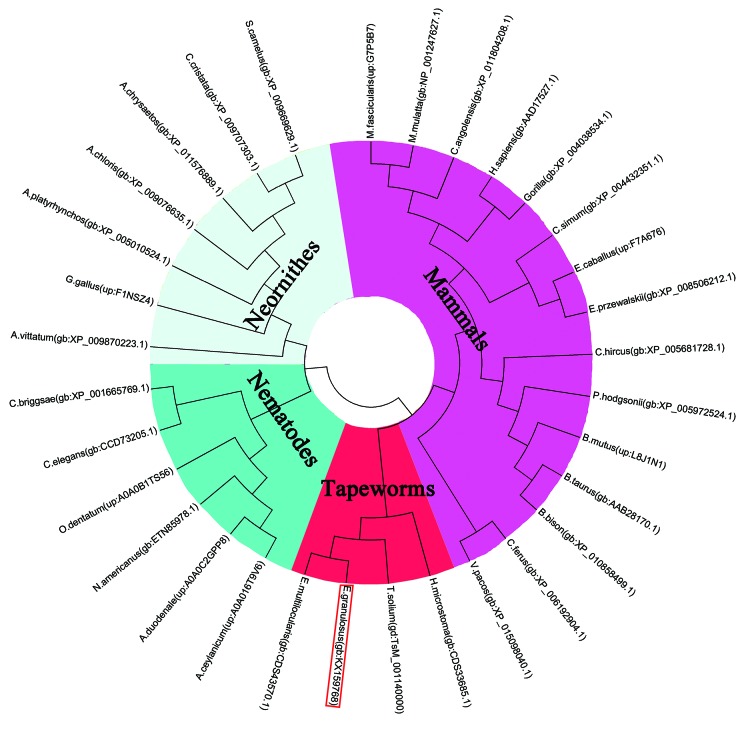
Phylogenetic analysis of leucine aminopeptidase protein from *Echinococcus granulosus* (Eg-LAP) with homologous leucine aminopeptidases. The phylogenetic tree was constructed based on the neighbor-joining method using MEGA v. 5.05. *E. granulosus*, *Echinococcus granulosus*; *E. multilocularis*, *Echinococcus multilocularis*; *T. solium*, *Taenia solium*; *H. microstoma*, *Hymenolepis microstomia*; *C. elegance*, *Caenorhabditis elegance*; *N. americanus*, *Necator americanus*; *A. ceylanicum*, *Ancylostoma ceylonicum*; *A. duodenale*, *Ancylostoma duodenale*; *C. briggsae*, *Caenorhabditis briggsae*; *O. dentatum*, *Oesophagostomum dentatum*; *G. gallus*, *Gallus gallus*; *A. chloris*, *Acanthisitta chloris*; *S. camelus*, *Struthio camelus*; *C. cristata*, *Cariama cristata*; *A. platyrhynchos*, *Anas platyrhynchos*; *A. vittatum*, *Apaloderma vittatum*; *A. chrysaetos*, *Aquila chrysaetos*; *B. taurus*, *Bos Taurus*; *B. bison*, *Bison bison*; *B. mutus*, *Bos mutus*; *C. hircus*, *Capra hircus*; *P. hodgsonii*, *Pantholops hodgsonii*; *V. pacos*, *Vicugna pacos*; *C. ferus*, *Camelus ferus*; *C. simum*, *Ceratotherium simum*; *E. caballus*, *Equus caballus*; *E. przewalskii, Equus przewalskii*; *H. sapiens*, *Homo sapiens*; *M. mulatta*, *Macaca mulatta*; *M. fascicularis*, *Macaca fascicularis*; *C. angolensis*, *Colobus angolensis*. gb, GenBank ID; gd, GeneDB ID; up, UniProt ID.

The cDNA sequence of Eg-LAP was 1824 bp encoding a 607-amino-acid protein. The protein had a predicted pI of 6.6, mass of 65.8 kDa, and the instability index (II) was 44.65. No transmembrane regions or signal peptide sequences were predicted in the deduced amino acid sequence. Comparing protein sequences between different species, we found that Eg-LAP shared 95.45% identity with *Echinococcus multilocularis* leucine aminopeptidase (E.m-LAP), 69.08% identity with *Taenia solium* leucine aminopeptidase (T.s-LAP), 32.25% identity with human leucine aminopeptidase (Hs-LAP), 21.04% identity with leucine aminopeptidase from *Caenorhabditis elegans* (Ce-LAP), 22.48% identity with leucine aminopeptidase from *Oesophagostomum dentatum* (Od-LAP), and 17.73% identity with Ovis aries leucine aminopeptidase (Oa-LAP). Sequence analysis was performed based on research into *Caenorhabditis elegans*, *Acanthamoeba castellanii*, and *Toxoplasma gondii* LAPs [27,28,29]. All the sequences contain a highly similar octapeptide (NTDAEGRL), which is characteristic of M17 aminopeptidases [30]. Highly similar amino acid residues were found for metal binding (Lys320, Asp325, Asp343, Asp402, Glu404) and catalytic site formation (Lys 332, Arg406). The homologous octapeptide contained two of the metal binding (Asp 402, Glu404) and one of the catalytic site forming (Arg406) residues [27,28,29] (Figure 1). Phylogenetic analysis showed the relationship of EgLAP with leucine aminopeptidase from other parasites and from mammals. EgLAP was clustered into a clade closely related to LAP from other cestode parasites such as *Echinococcus multilocularis*, *Taenia solium*, and *Hymenolepis microstoma* (Figure 2).

### 2.2. Expression and Purification of rEg-LAP

**Figure 3:**
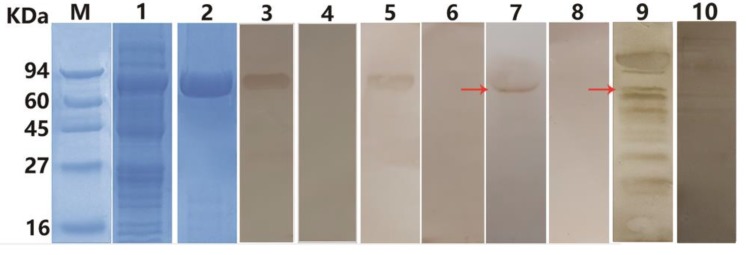
SDS-PAGE and Western blot analysis. Expression and purification of rEg-LAP and identification using serum from *E. granulosus*-infected sheep. M, molecular mass markers in KDa; lane 1, whole protein from *E. coli* BL21 (DE3) transformants containing pET28a(+)-Eg-LAP induced by IPTG; lane 2, sample after Ni2+ column purification of recombinant His-tagged protein; lane 3, purified rEg-LAP was probed with anti-rEg-LAP rabbit serum; lane 4, purified rEg-LAP was probed with native (preimmune) rabbit serum; lane 5, purified rEg-LAP was probed with serum from *E. granulosus*-infected sheep; lane 6, purified rEg-LAP was probed with uninfected sheep serum; lane 7, endogenous Eg-LAP from protoscoleces was probed with anti-rEg-LAP rabbit serum; lane 8, the total protein of PSCs was probed with native (preimmune) rabbit serum; lane 9, total protein extracts of protoscoleces was probed with serum from *E. granulosus*-infected sheep; lane 10, total protein extracts of protoscoleces was probed with uninfected sheep serum. Red arrow indicates the objective band.

*E. coli* BL21 (DE3) was induced to express insoluble rEg-LAP with a His-tag. Purified protein was examined by SDS-PAGE; the molecular mass was as expected (around 72 kDa including the His-tag) (Figure 3). Purified anti-rEg-LAP rabbit IgG was used to identify the native Eg-LAP protein from PSCs and purified rEg-LAP, which produced bands on western blots at 66 kDa and 72 kDa respectively. In Line 9, at 66 kDa also had a band we presume it as native LAP (Figure 3). Then, the rEg-LAP was recognized by the serum of *E. granulosus*-infected sheep.

### 2.3. Identification in Parasites and Fluorescence Immunohistochemistry

**Figure 4:**
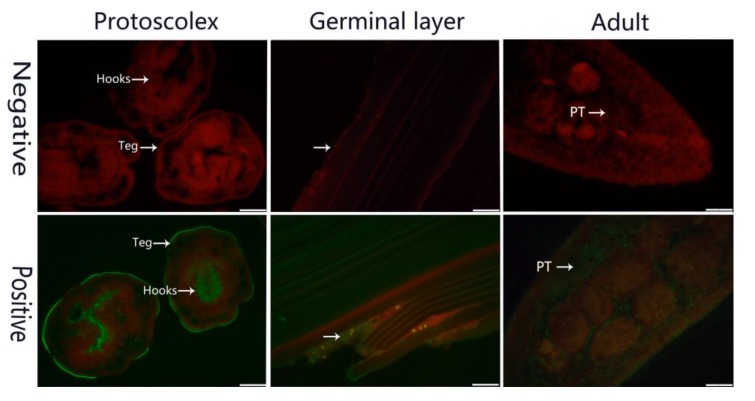
Immunofluorescence localization of Eg-LAP in all stages of *E. granulosus*. Eg-LAP in the protoscolex, germinal layer and adult worm labeled by incubation with specific anti-rEg-LAP IgG (positive), or preimmune serum (negative), then with FITC-conjugated anti-rabbit IgG. Teg, tegument; PT, parenchymatous tissue. Scale bars: 50 μm.

Fluorescence immunohistochemistry showed that native Eg-LAP was distributed in the tegument and hooks of PSCs, and widely distributed in the whole germinal layer and adult worm parenchymatous tissue. No signal was detected in the negative controls (Figure 4).

### 2.4. Establishment of Indirect ELISA

The optimal conditions for LAP antigen indirect ELISA were antigen concentration 1.4 μg/well and serum dilution 1:80 with an optimal P/N value of 2.80. In these conditions, twenty-four negative serum samples were tested: the mean absorbance was 0.3404, the standard deviation was 0.0516, and the cut-off value was therefore calculated as 0.4952 (Mean Abs + 3 SD). The optimal conditions for hydatid fluid antigen indirect ELISA were antigen concentration at 0.27 μg/well and serum dilution at 1:40.

### 2.5. Sensitivity, Specificity and Cross Reactivity Analysis of Indirect ELISA

**Figure 5:**
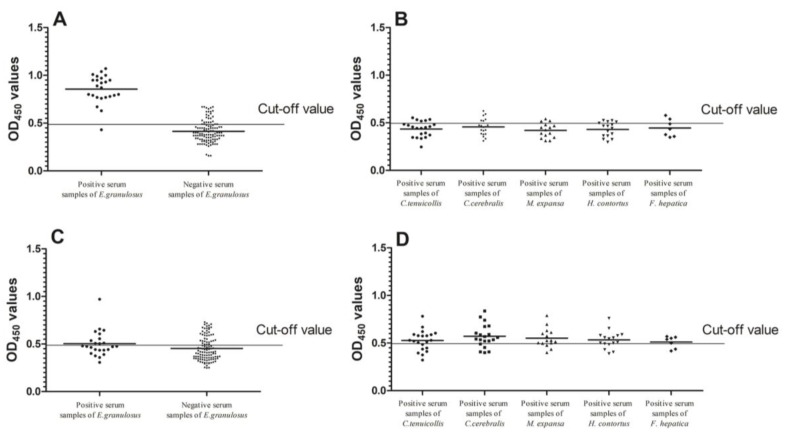
Specificity and sensitivity of the indirect ELISA. The cut-off value is 0.495. (**A**) The recombinant Eg-LAP specificity and sensitivity of indirect ELISA for *E. granulosus* diagnose; (**B**) The cross reactivity of rEg-LAP of indirect ELISA; (**C**) The hydatid cysts antigen specificity and sensitivity of indirect ELISA for *E. granulosus* diagnose; (**D**) The cross reactivity of hydaid cycts antigen of indirect ELISA.

The sensitivity of LAP indirect ELISA was 95.8% (23/24), while the specificity was 79.09% (87/110) (Figure 5A). We observed cross-reactivity with five positive serums against *C. tenuicollis*, six positive serums against *C. cerebralis*, three positive serums against *M. expansa*, four positive serums against *H. contortus* and two positive serums against *F. hepatica* (Figure 5B). There was statistical significance observed in the mean optical density (OD) value of *E. granulosus*-positive sera and other sera samples, including *C. tenuicollis*-positive sheep sera, *C. cerebralis*-positive goat sera, *M. expansa*-positive sheep sera, *H. contortus*-positive sheep sera, *F. hepatica*-positive sheep sera, and healthy rabbit sera (Mann–Whitney U, *z* = 7.636, *p* < 0.0001). There was statistical significance observed in the mean OD value of healthy sheep serum samples and the other positive serum samples (Mann–Whitney U, *z* = −2.064, *p* = 0.039). The sensitivity of hydatid fluid indirect ELISA was 41.7% (10/24), the specificity of hydatid fluid indirect ELISA was 65.45% (72/110) (Figure 5C). We observed cross-reactivity with 13 positive serums against *C. tenuicollis*, 15 positive serums against *C. cerebralis*, 11 positive serums against *M. expansa*, 11 positive serums against *H. contortus* and five positive serums against *F. hepatica* (Figure 5D). There were no differences between *E. granulosus*-positive sera and other sera samples, including *C. tenuicollis*-positive sheep sera, *C. cerebralis*-positive goat sera, *M. expansa*-positive sheep sera, *H. contortus*-positive sheep sera, *F. hepatica*-positive sheep sera, and healthy rabbit sera (Mann–Whitney U, *z* = 0.178, *p* = 0.859). There was statistical significance observed in the mean OD value of healthy sheep serum samples and the other positive serum samples (Mann–Whitney U, *z* = −5.036, *p* < 0.0001).

### 2.6. Reproducibility and Repeatability of Indirect ELISA

The coefficients of variation (CVs) of the interassay ranged from 0.13% to 5.28% (mean value = 1.85%) and those of the intra-assay ranged from 1.28% to 5.17% (mean value = 2.88%). All CVs were <10%, indicating that the rEg-LAP indirect ELISA was repeatable and reliable.

## 3. Discussion

LAPs are metallopeptidases, and the presence of Mn^2+^, Co^2+^, or Ni^2+^ improves their activity [27,31]. LAPs cleave N-terminal proteins and peptides and also play major roles in regulating cellular catabolism and anabolism [32,33]. LAPs are widely distributed in different organisms [27]. The M17 LAP monomers (53–55 kDa) assemble into homo-hexameric enzymes with high temperature (60–70 °C) and alkaline pH (8.5–9.5) optima [34,35,36]. M17 members topically contain the signature octapeptide NTDAEGRL [29,32]. This signature octapeptide is also present in the Eg-LAP sequence, which lacks the HEXXHX_18_E and GAMEN motifs of M1 peptidases. Previous research into LAP has focused on trematoda, but are still lacking in cestodes so far [37,38,39,40].

Studies on *F. gigantica* showed that Fg-LAP is only located on the surface of epithelial cells, where it is necessary for the digestion of peptides [41] *Paragonimus westermani* LAP is mainly distributed in the gut epithelium, where it is also responsible for digestion of proteins [42]. Aside from its presence in excretory-secretory products, *C. sinensis* LAP localizes to the tegument, as does *Schistosoma mansoni* LAP [38,40]. In this study, EgLAP locates on the germinal layer, the adult worms, the surface of PSCs tegument and the hooks of PSCs. Moreover, EgLAP was detected on the surface of the PSCs, which suggested that it might be involved in the absorption of nutrients and digestion of peptides, consistent with previous studies [41]. Of note, EgLAP showed up in the hooks of the PSCs suggested that it might be involved in fixation of the PSCs to the intestinal mucosa or interaction [43]. EgLAP is widely distributed in the germinal layer, suggesting that EgLAP may be indispensable in involving the formation of the germinal layer to and growth of PSCs [44]. EgLAP was also found in the parenchymatous tissue and tegument of adult worms which implies its functions in parasite interaction with the host, nutrition, immune evasion and modulation [38]. For example, LAPs in protozoan parasite *P. falciparum* has been confirmed to have roles in absorption of nutrients and immunoregulation and are further considered a potential drug target [45]. However, the function of EgLAP in parasite interaction with the host needs to be further verified.

Clinical symptoms are difficult to determine for *E. granulosus* infection, thus, an accurate diagnosis is important to control cystic echinococcosis in sheep. Ultrasound and other diagnostic methods have been used to improve the accuracy of diagnosis. However, compared with indirect ELISA, such methods have disadvantages of expense and scarce materials [13,14,16]. Moreover, compared with traditional methods, indirect ELISA has the advantages of high reproducibility and stability of antigen source. More importantly, compared with native antigens in the developed ELISAs, the recombinant antigen has higher purity and more stable reproducibility [17]. Therefore, to improve the immunodiagnosis of CE, establishment of an efficient indirect ELISA method is needed.

LAP of *Setaria cervi* [26], *Schistosoma japonicum* [24,46], and *Schistosoma mansoni* [25] have been reported as antigens in indirect ELISA to diagnose the human parasitic diseases. For *S. mansoni,* a sandwich-ELISA was established based on LAP antigen purified from the excretory/secretory material, which detected infected mice with 89.29% (50/56) sensitivity and 88.29% specificity. Use of the same method for infected humans showed sensitivity and specificity of 85% (51/60) and 80%, respectively [25]. LAP from *S. japonicum* could be used to detect a response 10 days after infection with a specificity of 98.1% (51/52) in acute patients and 87.8% (86/98) in chronic patients; thus, LAP-based ELISA is promising for the immunological diagnosis of *S. japonicum* patients [24,46]. In this study, we used rEg-LAP as the antigen to develop an indirect ELISA to diagnose cystic echinococcosis in sheep. The specificities of the rEg-LAP and hydatid fluid antigen-based indirect ELISA were 79.09% (87/110) and 65.45% (72/110), respectively, and the sensitivities were 95.8% (23/24) and 41.7% (10/24). Notably, the specificity and sensitivity of rEg-LAP were based on the small panel (110 negatives and 24 positives) tested samples; more serum sampling in filed is still needed to validate and improve the accuracy in future.

## 4. Materials and Methods

### 4.1. Ethics Statement

This study was reviewed and approved by the Care and Use of Laboratory Animals of the Animal Ethics Committee of Sichuan Agricultural University (Ya’an, China) (AECSCAU; Approval No. 2012-038, 7 February 2012). All animals were handled in strict accordance with the animal protection laws of the People’s Republic of China (a draft animal protection law was released on 18 September 2009) and the National Standards for Laboratory Animals in China (executed on 5 January 2002).

### 4.2. Animals and Parasites

Two New Zealand white rabbits (ten-weeks-old) were obtained from the Laboratory Animal Center of Sichuan Agricultural University. Cysts of *E. granulosus* were separated from infected sheep livers in Sichuan Province, China. All cysts were identified as *E. granulosus* sensu stricto (G1—G3) [47]. The hydatid fluid and protoscoleces (PSCs) were separated aseptically by centrifugation at 600× *g*. Adult worms were obtained from Department of Parasitology, Sichuan Agricultural University (collected from a dog 35 days post-infection with 20,000 PSCs).

### 4.3. Bioinformatics Analysis

The cDNA sequence of Eg-LAP was uploaded to GenBank (accession code: KX159768) (Available online: http://www.ncbi.nlm.nih.gov/). Open Reading Frame Finder (Available online: http://www.ncbi.nlm.nih.gov/gorf/gorf.html) and DNASTAR (Madison, WI, USA) were used to translate Eg-LAP gene sequence into amino acid sequence. Amino acid multiple sequence alignment was performed using ClustalX software version 1.83. A phylogenetic tree was established by the neighbor-joining (NJ) method with MEGA software (version 5.05). Signal sequences were predicted with SignalP (Available online: http://www.cbs.dtu.dk/services/SignalP/). Molecular weight (MW), isoelectric point (pI), conserved domains and protein properties were predicted using tools on the ExPaSy website (Available online: http://web.expasy.org/).

### 4.4. Expression and Purification of Recombinant EgLAP

Total RNA was extracted from PSCs using the RNA-prep Pure Tissue Kit (Tiangen, Beijing, China), and a Reverse Transcription System Kit (Thermo Fisher, Waltham, MA, USA) was used to synthesize first-strand cDNA. We designed the primers based on the sequence of EgrG_001133600 (Available online: http://www.genedb.org/Homepage). The expression sequence of Eg-LAP was amplified by PCR using primers 5′-CCGGAATTCATGTTTTCCAAAATGCGG-3′ and 5′-CCGCTCGAGTCAGACGTCTTCGTCCTCG-3′ with *Eco*RI and *Xho*I restriction enzyme sites (underlined), respectively. The PCR product was ligated into expression vector pET28a(+) (Novagen, Darmstadt, Germany) after digestion with *Eco*RI and *Xho*I. Then the recombinant plasmid was transformed into *Escherichia coli* BL21 (DE3) cells (Invitrogen, Carlsbad, CA, USA). Subsequently, transformants were induced with 0.8 mM isopropyl-β-d-1-thiogalactopyranoside (IPTG) for 6 h at 37 °C. The bacterial cells were centrifuged, resuspended in lysis buffer (100 mM NaCl, 50 mM NaH_2_PO_4_ (pH 8.0), 10 mM Tris–HCl (pH 8.0)), ultrasonicated, then centrifuged at 12,000× *g* for 10 min at 4 °C and collected inclusion bodies. The inclusion bodies were resuspended in lysis buffer plus 8 M urea and incubated on ice for 1 h to completely solubilize the protein. His-tagged rEg-LAP proteins were purified using Ni^2+^ affinity chromatography with His-affinity resin column (Bio-Rad, Hercules, CA, USA), and examined by 12% sodium dodecyl sulfate–polyacrylamide gel electrophoresis (SDS-PAGE). Protein concentrations were determined with a BCA Protein Assay Kit (NJJCBIO, Nanjing, China).

### 4.5. Sera

Positive serum samples were collected from farms in Sichuan Province (China), including 24 *E. granulosus*-infected (EI) sheep, 20 *Coenurus cerebralis*-infected sheep, 21 *Cysticercus tenuicollis*-infected goats, 15 *Moniezia expansa*-infected sheep, 16 *Haemonchus contortus*-infected sheep and 7 *Fasciola hepatica*-infected sheep. 134 negative sheep sera were collected from the cestode-free (CF) area and further confirmed by autopsy, 24 of them were used for calculated cut-off value. All sera were stored at −20 °C. Polyclonal antibody against Eg-LAP was prepared as in previous study [48]. Each rabbit was immunized subcutaneously with 200 μg recombinant Eg-LAP (rEg-LAP) emulsified with Freund’s complete adjuvant (Sigma, St. Louis, MO, USA), followed by three booster immunizations separately performed on days 14, 28 and 42. Rabbit anti-rEg-LAP serum was collected 2 weeks after the final immunization and the antibody titer was tested by ELISA. Then the anti-rEg-LAP serum was purified by HiTrap Protein A affinity chromatography (Bio-Rad).

### 4.6. Western Blotting (WB)

Recombinant Eg-LAP and the total protein extracts of PSCs were detected by 12% SDS-PAGE and then transformed onto a nitrocellulose membrane. After blocking with 5% skim milk, the membranes were incubated with infected sheep serum, or rabbit anti-rEg-LAP IgG (1:200) at 37 °C for 1 h and then incubated with rabbit anti-sheep or goat anti-rabbit IgG (H + L) HRP conjugate (1:2000, Bio-Rad) for 1 h at 37 °C. An Enhanced HRP-DAB Chromogenic Substrate Kit (Tiangen, Beijing, China) was used for detection. Non-infected sheep and pre-immunized rabbit sera were used as negative controls.

### 4.7. Immunolocalization

For immunohistochemical experiments, fresh PSCs, the germinal layer and adult worms were fixed in 4% paraformaldehyde-phosphate for 36 h, then embedded in paraffin and sliced into 4-μm thick sections. Sections were checked and selected under a microscope, deparaffinized in xylene, and dehydrated in ethanol. Next, sections were treated with 0.01 M citrate buffer for thermal remediation, and then 3% H_2_O_2_. After three washes, purified rabbit anti-rEg-LAP IgG (1:200 in PBS) was used to cover the sections overnight at 4 °C. After repeated washing steps, the sections were incubated with fluorescein isothiocyanate (FITC)-conjugated goat anti-rabbit IgG (H + L) (Bethyl Laboratories, Montgomery, TX, USA) at 37 °C for 1 h in darkness. Fluorescence was examined and photographed using a fluorescence microscope (OLYMPUS, IX71). Preimmune rabbit serum was used as the negative control.

### 4.8. Indirect ELISA

ELISAs were performed essentially as described [49,50]. Standard checkerboard titration was used to find the optimal conditions of antigen dilution and serum dilution for ELISA. Recombinant Eg-LAP and hydatid fluid antigen was two-fold serially diluted to eight concentrations from 5.6 μg/well to 0.04 μg/well and 4.32 μg/well to 0.033 μg/well respectively in coating buffer (carbonate-bicarbonate, pH 9.6). The 96-well ELISA plates were incubated with 100 μL antigen solution at 4 °C overnight. The plates were washed three times with PBS containing 0.05% Tween-20 then incubated for 1 h at 37 °C with blocking with 5% skim milk. After washing three times, serum samples serially diluted two-fold with PBS were added to the wells and incubated for 1 h at 37 °C. After an additional three washes, horseradish peroxidase (HRP)-conjugated rabbit anti-sheep or goat immunoglobulin G (Boster, Wuhan, China) (diluted 1:3000 in PBS) were added to the plates, then incubated at 37 °C for 1.5 h. Subsequently, after washing again with PBST, 3,3,5,5-tetramethylbenzidine (TMB, Tiangen, China) was added (100 µL/well). The reaction was stopped with 2 M H_2_SO_4_ after 15 min incubation in the dark at 37 °C. Results were read by a microplate reader (Thermo Fisher) at 450 nm. All optimal conditions were tested as per previous reports [51]. We chose the highest P/N value (between two ELISA well OD_450_ value ratio, the only difference was the positive and negative serum) experiment concentration as the optimal working condition. The indirect ELISA cut-off value was calculated by testing 24 negatives (CF) sheep serum samples, reading the mean OD_450_ value and adding three standard deviations (SD) of the OD_450_.

### 4.9. Sensitivity, Specificity, and Cross Reactivity of Indirect ELISA

Sensitivity = ELISA positive samples/true positive samples × 100%; Specificity = ELISA negatives samples/true negative samples × 100%. Twenty-four *E. granulosus*-positive (EI) sheep serum samples were used to evaluate the sensitivity. Specificity were evaluated by 110 negatives (CF) sheep serum samples. Serum samples from sheep infected with *C. cerebralis* (*n* = 20), goats infected with *C. tenuicollis* (*n* = 21), sheep infected with *M. expansa* (*n* = 15), sheep infected with *H. contortus* (*n* = 16) and sheep infected with *F. hepatica* (*n* = 7) were used as controls for detection the cross-reactivity of rEg-LAP.

### 4.10. Repeatability and Reproducibility of Indirect ELISA

All serum samples were used to evaluate the repeatability and reproducibility of the rEg-LAP and hydatid fluid antigen ELISA. Every sample was assessed for the intra-assay variability and interassay variability three times using coefficients of variation (CV).

### 4.11. Statistical Analysis

Statistical analyses were performed with Mann–Whitney test for comparison different groups using the IBM SPSS Statistics 20. *p*-values < 0.05 were considered to be statistically significant.

## 5. Conclusions

In this research, we found Eg-LAP sequence contain a signature octapeptide NTDAEGRL which inidicates that Eg-LAP belongs to M17 peptidase families. Based on immunolocalization of Eg-LAP, we found that Eg-LAP might be involved in some important biological functions. Its location on the surface and hooks of PSCs suggests it might be involved in fixation to the intestinal mucosa or interaction of the PSCs. Nevertheless, compared with previous studies involving the hydatid cyst antigen, the rEg-LAP-based indirect ELISA established in this study is more suitable for diagnosing and monitoring CE in the field.

## Abbreviations

rEg-LAP	recombinant *E. granulosus* leucine aminopeptidase
CE	Cystic echinococcosis
PSCs	Protoscoleces
CV	Coefficient of variation
EI	*E. granulosus*-infected
CF	cestode-free
